# Improving Melanoma Risk Stratification of Skin Color Type by Polygenic Scores of Skin Color and Melanoma

**DOI:** 10.1111/ijd.70121

**Published:** 2025-10-31

**Authors:** Huy Tran, Annabelle Ashworth, Zhuqing Shi, Jun Wei, S. Lilly Zheng, Ross M. Levy, Luzheng Liu, David Duggan, Aleksandar Sekulic, Jianfeng Xu

**Affiliations:** ^1^ Program for Genomic Translational Research, Endeavor Health Evanston Illinois USA; ^2^ Division of Dermatology, Department of Internal Medicine Endeavor Health Evanston Illinois USA; ^3^ Derick Dermatology Chicago Illinois USA; ^4^ Translational Genomics Research Institute, TGen – An Affiliate of City of Hope Phoenix Arizona USA; ^5^ City of Hope Duarte Arizona USA; ^6^ University of Chicago Pritzker School of Medicine Chicago Illinois USA

**Keywords:** Fitzpatrick scale, melanoma, polygenic score (PGS), skin color, skin of color, SOC

## Abstract

**Background:**

The Fitzpatrick skin type (FST) is commonly used in clinical settings to stratify melanoma risk across different skin types. However, it is subjective and does not capture intra‐type variability in risk. These limitations are particularly evident among lighter‐skinned individuals, who constitute the majority of those with European ancestry and generally have a higher melanoma risk.

**Methods:**

We assessed whether supplementing FST with validated polygenic scores for skin color (PGS_SC_) and melanoma (PGS_M_) improves risk stratification in 479,607 UK Biobank participants. Melanoma prevalence in individuals by FST, PGS_SC_, and PGS_M_ was estimated, and their differences were tested, adjusting for age, gender, and genetic background (top 10 principal components).

**Results:**

FST distribution was as follows: Very fair (7.77%), fair (68.06%), light olive (18.56%), olive (1.87%), brown (2.96%), and black (0.78%), with corresponding melanoma prevalence of 1.94%, 1.31%, 0.70%, 0.45%, 0.15%, and 0% (*P*_−trend_ < 0.001). Notably, PGS_SC_ significantly differentiated melanoma prevalence within each lighter‐skinned type (very fair, fair, and light olive), *P*_−trend_ < 0.001. Among lighter‐skinned individuals, the melanoma prevalence ranged widely across the lowest to highest deciles of PGS_SC_ (0.62%–2.12%). In addition, PGS_M_ also differentiated risk, with prevalence ranging from 0.39%–2.08% between low‐ and high‐risk groups. Among darker skin types (olive and brown), only PGS_M_ significantly differentiated melanoma risk, with prevalence ranging from 0.13% to 0.80% between low‐ and high‐risk groups.

**Conclusions:**

Integrating PGS of skin color and melanoma with the conventional FST classification significantly improves melanoma risk stratification and addresses key limitations of the FST, which, while widely used, lacks sensitivity to intra‐type risk variation.

## 1 Introduction

Melanoma is the fifth most common cancer diagnosed in the United States. In 2025, approximately 104,960 new cases of melanoma are expected to be diagnosed, and 8430 deaths are anticipated due to the disease [1, 2]. The 5‐year survival rate is significantly higher for patients with Stage I melanoma (98%) compared to those with Stage IV melanoma (35%), underscoring the importance of early diagnosis through screening to reduce melanoma‐related mortality [2]. While melanoma screening for the general population is not considered standard care, it is recommended for high‐risk individuals, including those with fair skin, a history of sunburns, a personal or family history of melanoma, and the presence of atypical moles [3, 4, 5, 6].

Lighter skin color is a major risk factor for melanoma, due to low melanin levels and higher proportions of pheomelanin, both of which result in lowered protection against DNA damage caused by ultraviolet (UV) radiation. The Fitzpatrick skin type (FST) scale, comprising Type I (very fair), Type II (fair), Type III (light olive), Type IV (olive), Type V (brown), and Type VI (black), is widely used in clinical settings for melanoma risk stratification [7, 8]. The ability to tan and propensity to burn are the key determinants of the Fitzpatrick scale. Although the FST scale can be classified via self‐report or visual inspection and is effective at differentiating melanoma risk across skin types, it is subjective and fails to capture risk variability within each skin type. This is particularly evident among lighter‐skinned individuals (very fair, fair, and light olive), who represent the majority of those of European ancestry and are generally considered at elevated risk [9]. Therefore, more accurate and personalized risk assessment tools for melanoma are needed.

Skin color is a polygenic trait, and hundreds of thousands of skin color‐associated single‐nucleotide polymorphisms (SNPs) have been identified through genome‐wide association studies (GWAS) [10, 11, 12, 13, 14]. The polygenic score (PGS) for skin color (PGS_SC_), calculated from these skin color‐associated SNPs, provides an objective and informative stratification tool for skin color and assessing melanoma risk [14, 15, 16]. As a continuous variable, PGS_SC_ offers a personalized genetic risk assessment for every individual. Additionally, many risk‐associated SNPs for melanoma have been identified through GWAS [17, 18, 19]. While there is considerable overlap between SNPs associated with skin color and risk for melanoma, the majority of these melanoma risk‐associated SNPs are independent of skin color. These SNPs are likely involved in other mechanisms such as cell growth and DNA repair [20]. The PGS for melanoma (PGS_M_), derived from these SNPs, has been demonstrated to be an informative tool for stratifying melanoma risk [21, 22].

Given the genetic basis of skin color and melanoma, we hypothesized that adding these PGS tools to the phenotypic features captured by FST could provide an enhanced and more accurate approach to predicting melanoma risk at a personalized level.

## 2 Methods

We tested this hypothesis using the UK Biobank (UKB) data, a population‐based cohort of participants aged between 40 and 69 years old at recruitment from across the United Kingdom (accessed under application number: 50295) [23]. Diagnosis of melanoma was based on the International Classification of Diseases (ICD)‐10 code C43, C43.0–C43.9 from medical records and cancer registry. The FST scale was derived from a self‐reported questionnaire on untanned skin color (data‐field 1717) at recruitment and mapped to the standard six‐type FST scale (Type I–VI). Genome‐wide SNPs were genotyped using SNP arrays.

Genetic ancestry probabilities for each individual were estimated using principal components (PCs) derived from 16,309 ancestry‐informative markers distributed across the genome. Ancestral populations included Finnish (FIN), non‐Finnish European (NFE), Ashkenazi Jewish (ASJ), East Asian (EAS), African (AFR), Admixed American (AMR), and South Asian (SAS). Individuals were assigned to a specific ancestry group if their predicted probability for that ancestry exceeded 75%. For downstream analyses, participants were classified as having European ancestry if > 75% of their genetic ancestry was FIN, NFE, and/or ASJ, and as non‐European ancestry otherwise.

Pan‐ancestry PGSs for skin color and melanoma were selected from published methods in the PGS Catalog [15, 24], including PGS_SC_ (PGS002110, with 275,831 SNPs [25]) and PGS_M_ (PGS003382, 672 SNPs [22]). The majority of the SNPs in PGS_M_ (89%) did not overlap with those of PGS_SC_, although the two PGSs were correlated due to shared SNPs (*N* = 58), *r* = 0.52, *p* < 0.001. Raw PGS values for each participant were calculated from downloaded SNP weights and imputed genotypes. Ancestry‐adjusted PGS was then calculated using the first four PCs, as described previously [26]. PGS values were also categorized into deciles for PGS_SC_ and three risk groups for PGS_SC_ based on percentile cutoffs corresponding to relative risks (RRs) of 0.5 and 1.5 for melanoma compared to the entire cohort: low (< 0.5), average (0.5–1.49), and high (≥ 1.5).

For ease of presentation and interpretation, the primary measure was melanoma prevalence across FST categories and genetic risk strata of PGS_SC_ and PGS_M_. To reduce potential confounders of age, age‐standardized prevalence (ASP) was also calculated using the age distribution (< 60, 60–69, 70–79, and > 80 years) of the entire UKB. Differences in prevalence across ordered skin type risk categories were tested using logistic regression with a linear trend term (i.e., test for trend), adjusting for age, gender, and genetic background (top 10 PCs).

In addition, more formal statistical tests were performed to compare several prediction models using Cox proportional hazards with age as the underlying time scale. We evaluated the following models (each additionally adjusted for sex and PCs): FST‐only, PGS_SC_‐only, PGS_M_‐only, PGS_SC_ + PGS_M_, and the full model (FST + PGS_SC_ + PGS_M_). Proportional hazards assumptions were assessed, and no violations were detected. Model comparison used likelihood‐ratio tests for nested models. Calibration was evaluated by calibration slope and by calibration plots of observed cumulative incidence versus predicted risk at age 75 years. Discrimination was quantified by Harrell's C‐index; differences in C‐index were tested via bootstrap (1000 resamples). Improvement of genetic models over the FST‐only model was assessed using net reclassification improvement (NRI) to assess if they better reclassify individuals into more appropriate risk categories for an event.

## 3 Results

A total of 479,607 participants were included in this study after excluding individuals with more than 5% missing SNP data and those with unknown skin type (self‐reported skin color responses such as “Do not know” or “Prefer not to answer”) (Table 1). The median age at last follow‐up was 71 years (interquartile range [IQR], 64–77), 45.6% were male, and 94.2% were of European ancestry. Overall, 5681 participants (1.18%) had a diagnosis of melanoma.

**TABLE 1:** Characteristics of study subjects, Fitzpatrick skin type (FST), and melanoma risk.

	All	Very fair	Fair	Light olive	Olive	Brown	Black
Subjects, No. (%)	479,607 (100.00)	37,253 (7.77)	326,429 (68.06)	89,011 (18.56)	8980 (1.87)	14,205 (2.96)	3729 (0.78)
Age at end follow‐up, years, median (IQR)	71 (64–77)	71 (63–76)	72 (65–77)	71 (63–76)	70 (63–76)	66 (60–74)	65 (59–72)
Gender, male, No. (%)	218,902 (45.64)	13,914 (37.35)	152,189 (46.62)	38,097 (42.80)	5230 (58.24)	7700 (54.21)	1772 (47.52)
Ancestry, No. (%)*							
European							
NFE	435,031 (90.71)	35,712 (95.86)	312,951 (95.87)	77,907 (87.53)	6650 (74.05)	1782 (12.54)	29 (0.78)
FIN	691 (0.14)	70 (0.19)	473 (0.14)	137 (0.15)	8 (0.09)	3 (0.02)	0 (0.00)
ASJ	2954 (0.62)	155 (0.42)	1543 (0.47)	1136 (1.28)	110 (1.22)	10 (0.07)	0 (0.00)
Mixed European	13,236 (2.76)	961 (2.58)	7736 (2.37)	4091 (4.60)	355 (3.95)	88 (0.62)	5 (0.13)
Non‐European							
AFR	5306 (1.11)	22 (0.06)	200 (0.06)	35 (0.04)	101 (1.12)	2311 (16.27)	2637 (70.72)
AMR	766 (0.16)	25 (0.07)	167 (0.05)	314 (0.35)	73 (0.81)	179 (1.26)	8 (0.21)
EAS	1918 (0.40)	25 (0.07)	492 (0.15)	756 (0.85)	145 (1.61)	494 (3.48)	6 (0.16)
SAS	8970 (1.87)	118 (0.32)	1246 (0.38)	946 (1.06)	569 (6.34)	6023 (42.40)	68 (1.82)
Mixed non‐European	10,735 (2.24)	165 (0.44)	1621 (0.50)	3689 (4.14)	969 (10.79)	3315 (23.34)	976 (26.17)
Melanoma prevalence							
Observed, No. (%)	5681 (1.18)	724 (1.94)	4275 (1.31)	621 (0.70)	40 (0.45)	21 (0.15)	0 (0.00)
Age‐standardized (95% CI)	1.19 (1.15–1.22)	1.97 (1.83–2.11)	1.30 (1.26–1.33)	0.72 (0.66–0.77)	0.47 (0.32–0.61)	0.17 (0.09–0.25)	0.00 (0.00–0.00)

Abbreviations: ASJ, Ashkenazi Jewish; AFR, African; AMR, Admixed American; CI, confidence interval; EAS, East Asian; IQR, interquartile range; NFE, non‐Finnish European; FIN, Finnish; SAS, South Asian.

* Subjects with probability of > = 0.75.

We first tested the association between melanoma risk and the FST scale. The FST distribution in the cohort was: very fair (7.77%), fair (68.06%), light olive (18.56%), olive (1.87%), brown (2.96%), and black (0.78%) (Table 1). Melanoma prevalence varied significantly across the six skin types and decreased from lighter to darker skin types: very fair (1.94%), fair (1.31%), light olive (0.70%), olive (0.45%), brown (0.15%), and black (0%), *P*_−trend_ < 0.001. ASP showed a similar gradient after standardizing age distributions. However, FST was not able to capture variability in melanoma risk within each skin type. This limitation was particularly pronounced for lighter‐skinned individuals (very fair, fair, and light olive), who comprise the majority of the cohort and are generally at elevated risk. For example, 68.06% of participants reported fair skin type, yet FST would assign them a uniform melanoma risk (1.31% average prevalence) despite known variability in their risk of melanoma.

**FIGURE 1:**
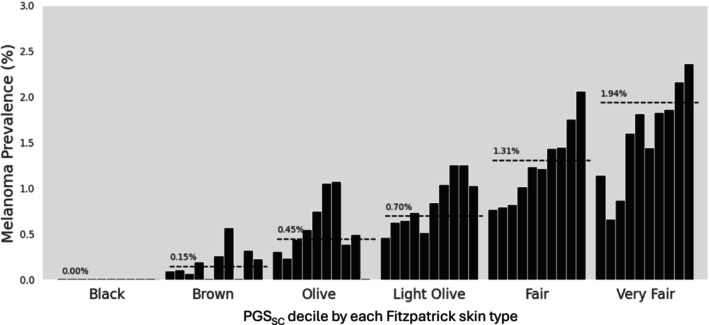
Observed melanoma prevalence by PGS_SC_ decile in each Fitzpatrick skin type (FST). The horizontal dotted lines indicate the average melanoma prevalence of each FST. Detailed sample size and prevalence (observed and age‐standardized) are presented in Table S1.

To address this limitation, we assessed whether genetic susceptibility to melanoma within each skin type can be better refined by PGS for skin color. The observed melanoma prevalence increased significantly with higher PGS_SC_ deciles within each of the lighter‐skinned types (very fair, fair, and light olive); *P*_−trend_ < 0.001 after adjusting for age, gender, and genetic background (top 10 PCs) (Figure 1, Table S1). For individuals of darker skin types, however, this trend was weaker; *P*_−trend_ was 0.04 and 0.03 for olive and brown types, respectively.

To further differentiate genetic susceptibility to melanoma beyond factors influencing skin color and melanin levels, we evaluated the performance of PGS_M_ in melanoma risk stratification. PGS_M_, as a continuous variable, was significantly associated with melanoma risk in both lighter‐skinned (*p* < 0.001) and darker‐skinned individuals (*p* < 0.001). When categorized, PGS_M_ also effectively stratified melanoma risk (Table 2). For those with low, average, and high‐risk PGS_M_ groups, respectively, observed melanoma prevalence was 0.39%, 0.99%, and 2.08% in the lighter‐skinned individuals and 0.13%, 0.23%, and 0.80% in the darker‐skinned individuals, both *P*_−trend_ < 0.001.

**TABLE 2:** Melanoma prevalence by PGS melanoma (PGS_M_) among different skin types.

Group	Observed melanoma prevalence (%)		Age‐standardized prevalence (95% CI)	
	Lighter skin (Type I‐III)	Darker skin (Type IV—V)	Lighter skin (Type I‐III)	Darker skin (Type IV—V)
Overall	5620/452,693 (1.24)	61/23,185 (0.26)	1.24 (1.21–1.27)	0.29 (0.22–0.37)
PGS_M_ risk group				
Low	132/33,832 (0.39)	5/3758 (0.13)	0.39 (0.32–0.45)	0.14 (0.01–0.26)
Average	2925/295,776 (0.99)	39/17,312 (0.23)	0.99 (0.95–1.02)	0.25 (0.17–0.33)
High	2563/123,085 (2.08)	17/2115 (0.80)	2.07 (2.00–2.15)	0.87 (0.46–1.28)

Abbreviations: 95% CI, 95% confidence interval; PGS_M_, polygenic scores of melanoma.

**FIGURE 2:**
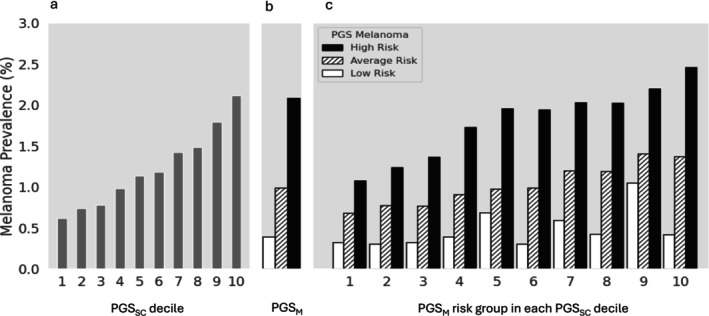
Observed melanoma prevalence among lighter‐skinned individuals by PGS_SC_ decile (a), PGS_M_ risk group (b), and the combination of both PGSs (c). For PGS_M_ risk group, open, slashed, and solid bars represent low‐, average‐, and high‐risk groups, respectively. Detailed sample size and prevalence (observed and age‐standardized) are presented in Table S2.

**FIGURE 3:**
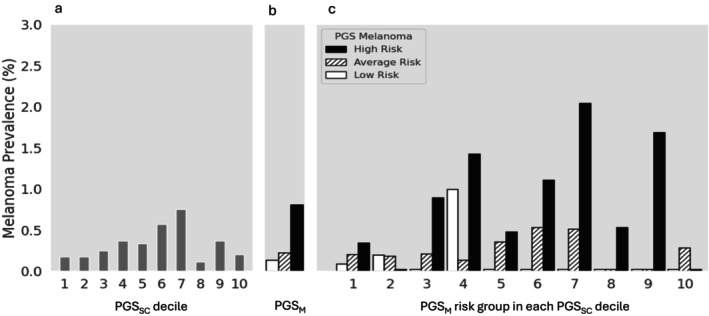
Observed melanoma prevalence among darker‐skinned individuals by PGS_SC_ decile (a), PGS_M_ risk group (b), and the combination of both PGSs (c). For PGS_M_ risk group, open, slashed, and solid bars represent low‐, average‐, and high‐risk groups, respectively. Detailed sample size and prevalence (observed and age‐standardized) are presented in Table S2.

Notably, PGS_M_ further refined melanoma risk stratification by PGS_SC_ (Table S2). Among lighter‐skinned individuals (very fair, fair, and light olive), where melanoma prevalence was strongly associated with PGS_SC_ decile (Figure 2a), PGS_M_ provided additional differentiation within each PGS_SC_ decile (Figure 2b,c), *P*_−trend_ < 0.001. Individuals with both the highest PGS_SC_ decile and high‐risk PGS_M_ had the highest melanoma prevalence (2.46%), while those with low‐risk PGS_M_ had generally low melanoma prevalence regardless of PGS_SC_ decile. Among individuals with darker skin types (olive and brown), where PGS_SC_ deciles were weakly associated with melanoma risk (Figure 3a), PGS_M_ provided a valuable risk differentiation tool (Figure 3b,c). Those in the high‐risk PGS_M_ group typically had higher melanoma prevalence, especially with higher PGS_SC_ deciles. For example, the melanoma prevalence was 2.04% and 1.69% for individuals in the 7th and 9th PGS_SC_ deciles.

Finally, comparisons of several Cox regression models further support the added value of PGS_SC_ and PGS_M_ beyond FST for predicting time to develop melanoma and discriminating risk (Table S3). Relative to the FST‐only model, the models with PGS_SC_‐only, PGS_M_‐only, and the full model (FST, PGS_SC_, and PGS_M_) had significantly better performance (higher C‐index, positive NRI, and higher calibration coefficient). For example, the C‐index increased from 0.56 (95% CI, 0.54–0.58) for FST‐only to 0.62 (0.61–0.63) for PGS_SC_‐only, 0.64 (0.63–0.66) for PGS_M_‐only, and 0.65 (0.63–0.67) for the full model (*p* < 0.001 for all ΔC‐index). A likelihood‐ratio test also indicated that the full model fit significantly better than the nested FST‐only model (*p* < 0.001).

## 4 Discussion

In this large population‐based cohort, we confirmed the excellent performance of FST in differentiating melanoma risk across skin types [7, 8], but also highlighted its key limitation of inability to differentiate variability in melanoma risk within each skin type. This limitation is particularly pronounced for lighter‐skinned individuals (very fair, fair, and light olive), who comprise the majority of the cohort and are generally at elevated risk. Importantly, we showed that melanoma risk among lighter‐skinned types can be effectively differentiated using both PGS_SC_ and PGS_M_. For individuals with darker skin types (olive and brown), PGS_M_ significantly differentiated melanoma risk from melanoma prevalence. These results suggest that genetic risk for lighter‐skinned individuals can be measured by variants related to skin color and other mechanisms (e.g., cell growth and DNA repair). In contrast, among darker‐skinned individuals, variation of skin color plays a lesser role in melanoma risk, and only variants related to non‐skin color mechanisms are informative for differentiating their risk.

Another major observation was the crossover of melanoma prevalence between lighter‐ and darker‐skinned individuals when stratified by PGS_M_ groups. Darker‐skinned individuals in the high‐risk PGS_M_ group had significantly higher melanoma prevalence (0.80%) than lighter‐skinned individuals in the low‐risk PGS_M_ group (0.39%), *p* < 0.001. The former represented 9% of darker‐skinned individuals, while the latter accounted for 7% of lighter‐skinned individuals.

We recognized that these findings, while statistically robust, yield modest improvements in risk stratification. Nevertheless, these improvements are clinically meaningful, particularly relative to FST alone. Among lighter‐skinned individuals who are generally considered high risk, melanoma prevalence was 2.46% in those with the highest PGS_SC_/PGS_M_ combination and 0.33% in those with the lowest, corresponding to a 2.13% absolute difference. This magnitude is larger than the difference (0.98%) observed between lighter‐ and darker‐skinned individuals in the UKB (melanoma prevalence 1.24% vs. 0.26%, respectively). Because the lighter–darker skin difference already informs screening recommendations, a similarly sized gradient by genetic risk is clinically appreciable, especially within the large subgroup of lighter‐skinned individuals who are generally considered high risk yet exhibit substantial heterogeneity in genetic susceptibility.

The improved and more personalized melanoma risk stratification by PGSs over FST has important clinical implications for prevention and screening among both lighter‐ and darker‐skinned individuals. It could empower individuals, particularly younger people with high genetic risk, to make lifestyle modifications for minimizing their UV exposures. Given that melanoma is multifactorial, arising from interactions between genetic susceptibility and environmental exposures [27], it is particularly important for individuals with high genetic predisposition to minimize UV exposure (including avoidance of indoor tanning), practice rigorous photoprotection (e.g., broad‐spectrum sunscreen use, protective clothing/shade), and administer regular self–skin examination. While relative risk reductions from photoprotection are likely similar across PGS strata, absolute risk reductions should be larger among high‐PGS individuals due to their higher baseline risk. It also has the potential to help dermatologists and other health care providers in determining the appropriate frequency of full‐body skin examinations (FSE) based on individual risk levels. Currently, there is no consensus on how often FSE should be performed in individuals without a skin cancer history [3, 4, 5, 6]. While most studies neither recommend nor are against routine FSE due to insufficient evidence on its effectiveness in reducing melanoma mortality, they do indicate that high‐risk individuals may benefit from vigilant skin examination [28]. This personalized risk‐based approach is especially relevant given the ongoing shortage of dermatologists in the United States and other countries.

A clinical implementation framework is needed to translate PGS_SC_/PGS_M_ into practice, including (1) performing external validation to confirm findings in independent cohorts, with particular attention to non‐European ancestry subgroups, (2) developing consensus thresholds and corresponding screening strategies that align with current recommendations, (3) offering education to providers (dermatologists and primary care clinicians) as well as patients on the complementary role of PGS alongside FST, including limitations, interpretation, and equity considerations, and (4) implementing structured ordering and standardized reporting of PGS_SC_/PGS_M_ in the electronic health record (EHR), with clinical decision support and clear, patient‐friendly report language.

Several limitations of this study should be noted. First, our primary objective was to evaluate how genetic testing can complement the FST scale to enable more personalized melanoma risk stratification. We adopted this FST‐centered framework because FST is already widely used in clinical settings and thus is highly relevant for dermatologists and primary care physicians. However, FST has well‐documented limitations, including its subjective nature, difficulty in reliably distinguishing skin types, Eurocentric bias, underrepresentation of diverse skin tones, and potential for misinterpretation or misuse in clinical practice [29, 30, 31]. Accordingly, we acknowledge that an FST‐centered framework may not represent the optimal model for melanoma risk assessment. An alternative genetics‐centered approach, without requiring initial FST classification, may address many of these limitations. Second, although our cohort included approximately 27,000 individuals with darker skin and/or non‐European ancestry, the sample size for these subgroups was relatively small compared to lighter‐skinned individuals of European ancestry. Larger studies are needed to obtain more robust estimates of melanoma prevalence and genetic risk in underrepresented populations. Third, the performance of the PGS_M_ for melanoma risk stratification in the current study may be subject to overfitting, as 57% of participants in our study were included in the development of the PGS [22]. This concern is less applicable to PGS_SC_, which, although partially developed using UK Biobank data [15], was not trained or optimized using melanoma outcomes. Nevertheless, independent validation of our findings in external populations is essential.

In conclusion, this study demonstrates that integrating PGS for skin color and melanoma with the conventional FST classification significantly improves melanoma risk stratification in both lighter‐ and darker‐skinned individuals.

## Conflicts of Interest

Endeavor Health has agreements with GenomicMD and GoPath Laboratories for genetic tests of polygenic risk scores. J. Xu serves as a scientific advisory board member for GoPath Diagnostics and GenomicMD.

## Supporting information


**Table S1:** Melanoma prevalence by PGS skin color (PGS SC) among different Fitzpatrick skin types.
**Table S2:** Melanoma prevalence by PGS melanoma (PGSM) among different skin types.
**Table S3:** Cox proportional hazard analysis for various models.

## Data Availability

The data that support the findings of this study are available from the corresponding author upon reasonable request.
